# Maximal discharge rate of motor units determines the maximal rate of force development during ballistic contractions in human

**DOI:** 10.3389/fnhum.2014.00234

**Published:** 2014-04-22

**Authors:** Jacques Duchateau, Stéphane Baudry

**Affiliations:** Laboratory of Applied Biology, ULB Neurosciences Institute, Université Libre de BruxellesBrussels, Belgium

**Keywords:** fast contraction, motor neuron discharge rate, rate of force development, training, ageing

## Introduction

The magnitude of the neural activation, and hence the force produced by a muscle, depend on the number of motor units activated (recruitment) and the rates at which motor neurons discharge action potentials (rate coding). Although the recruitment order of motor units (size principle) is similar for contractions during which the force is gradually increased (ramp contraction) and those during which the force is produced as fast as possible (see Duchateau and Enoka, 2011), rate coding differs between the two types of contractions (Desmedt and Godaux, 1977a,b; Bawa and Calancie, 1983). Motor unit discharge rate increases progressively during slow ramp contractions (Milner-Brown et al., 1973) whereas fast contractions involve high instantaneous discharge rate that decreases thereafter (Desmedt and Godaux, 1977a; Van Cutsem et al., 1998). Maximal discharge rate during slow isometric ramp contractions usually reaches values of 20–50 Hz whereas it can attain much higher values (>100 Hz), albeit briefly, during fast contractions (for reviews, see Enoka and Fuglevand, 2001; Duchateau and Enoka, 2011)

Fast isometric contractions can be performed in different ways. A first possibility is to increase force as quickly as possible up to a certain level and to maintain this force for a few seconds (step and hold contraction). An alternative way is to produce force as fast as possible but to relax the muscle immediately after the target force is reached. Such impulse-like contractions have been termed *ballistic contractions* (Desmedt and Godaux, 1977a). Although both contractions involved reaching a target force as fast as possible, results from our laboratory indicate that the maximal rate of torque development is ~16% greater for ballistic than step and hold contractions (465.2 ± 17.4 vs. 400.5 ± 20 Nm/s; mean ± SD) performed with the ankle dorsiflexor muscles. Considering the difference in motor unit discharge rate between slow and fast contractions, these data suggest that ballistic contractions could be used to assess the maximal discharge rate of motor neurons in humans.

## Motor unit discharge rate during ballistic contraction

Desmedt and Godaux (1977a) were the first to provide a detailed description of motor unit discharge in the tibialis anterior muscle during ballistic contractions. They reported that during ballistic contractions, motor units usually began to discharge at high instantaneous rates (60–120 Hz) that thereafter declined progressively during their successive discharges, presumably reflecting the initial phase of discharge rate adaptation observed during repetitive activation of motor neurons (Sawczuk et al., 1995; Miles et al., 2005). Such discharge pattern has been also reported for the first dorsal interosseus (Desmedt and Godaux, 1977b) and the masseter (Desmedt and Godaux, 1979), with very brief interspike interval (<10 ms) mainly observed for the initial discharges (Desmedt and Godaux, 1977a; Van Cutsem et al., 1998; Van Cutsem and Duchateau, 2005). Similar brief interspike intervals have also been recorded in the flexor carpi radialis during fast and hold contractions (Bawa and Calancie, 1983). Such high motor unit discharge rates are similar to those reported for motor neurons in animal studies in response to fast current injection (Kernell, 1965; Baldissera et al., 1987; Sawczuk et al., 1995), and should mainly reflect the effect of the strong excitatory inputs required to produce ballistic contractions. However, these very high discharge rates could also be influenced by the trajectory of motor neuron membrane after de repolarization phase (delayed depolarization phase and/or after-hyperpolarization period—AHP) at the time of the activation (see Garland and Griffin, 1999; Kudina and Andreeva, 2013).

## Task-related changes in discharge rate

The discharge characteristics of single motor units during ballistic contractions can be modulated by the conditions under which the action is performed. For example, it has been observed that when a ballistic contraction with the ankle dorsiflexors was superimposed on a submaximal isometric contraction (20–25% of maximal force), the average discharge rate for the first three interspike intervals was significantly reduced by 22% (89.8 ± 14.6 vs. 115 ± 20.9 Hz; mean ± SD) compared with ballistic contractions performed from a resting state (Van Cutsem and Duchateau, 2005). The percentage of motor units that exhibited discharges rate above 200 Hz at the onset of the activation was also diminished (6.2 vs. 15.5%). Interestingly, the instantaneous discharge for the first interspike interval was much reduced (−37%) than the second (−18%) and third (−8%) intervals. This lower motor unit discharge rate during superimposed ballistic contractions was accompanied by a decrease in the maximal rate of force development (~16%). The slower rate of force development and reduced motor unit discharge rate during the superimposed ballistic contractions are, however, abolished when a brief silent period (usually called “premotor silent period”) was observed at the transition between the pre-activation (sustained contraction) and ballistic actions (Van Cutsem and Duchateau, 2005). A similar observation has been reported when a brief voluntary agonist relaxation (deactivation) was inserted between the sustained and the ballistic action (Duchateau and Baudry, 2012). These silent periods (unintentional and voluntary) are thought to enable motor neurons to achieve a non-refractory state leading to a more synchronous recruitment and a greater discharge rate of motor units during the subsequent ballistic action (Tsukahara et al., 1995; Van Cutsem and Duchateau, 2005). The changes in maximal discharge rate achieved during ballistic contractions with initial conditions likely reflect the history-dependent changes of motor neuron excitability (Heckman and Enoka, 2012), and on a functional point of view supports the association between the maximal motor unit discharge rate and the rate of force development.

## Long-term changes in discharge rate

A way to further investigate this association consists of studying long-term changes in the maximal discharge rate of human motor units, such as those occurring in response to training and ageing. For example, Van Cutsem et al. (1998) reported that 3 months of ballistic contractions of the ankle dorsiflexor muscles against a moderate load (30–40% MVC) enhanced the maximal rate of force development by 82% during ballistic contractions. Although no change was observed in the recruitment order of motor units, the average discharge rate of the first four action potentials increased by 38% after training (96.3 ± 39.5 vs. 69.9 ± 30.8 Hz; mean ± SD). The increase in discharge rate was significantly less for the first (+86%) and second (+70%) than the third (+124%) interspike intervals. In addition, training increased the number of motor units (5–33%) exhibiting discharges above 200 Hz at the onset of activation. Because the average time to peak force of motor unit mechanical responses was not significantly modified, the increase in the rate of force development during the ballistic contractions was mainly due to adaptation in motor unit discharge rate. Potential mechanisms that may explain the changes in motor unit discharge rate should involve different loci along the corticospinal pathway. Although some of these changes can occur at supraspinal level (Schubert et al., 2008), part of the adaptations presumably involve changes in the intrinsic properties of motor neurons, as observed after endurance training in rats (Gardiner et al., 2006).

In contrast to training, the ageing process induces a decline in the speed-related capacity of individuals. For example, the maximal rate of force development during ballistic contractions performed with the ankle dorsiflexor muscles was significantly lower by 48% in elderly (71–84 year) than in young adults (~20 year) (Klass et al., 2008). This age-related change was accompanied by a clear decline in the average motor unit discharge rate. As the decrease was less pronounced for the first (−19%) than for the second (−28%) and third (−34%) interspike intervals, this means that the aged motor units cannot sustain a high discharge rate during successive discharges. In addition, the percentage of motor units that exhibited initial discharges above 200 Hz was reduced (−45%) in elderly compared with young adults. As the rate of force development during electrically evoked contractions, that by-pass motor neurons activation, is less reduced than those during ballistic voluntary contractions, the decline in maximal motor unit discharge rate should significantly contribute to limit the performance of fast voluntary contractions with ageing. The age-related prolongation in the duration of motor neuron after hyperpolarization, as observed in the human biceps brachii by Piotrkiewicz et al. (2007), could be a relevant candidate to explain, at least in part, the reduced maximal rate of motor unit discharge during ballistic contractions in elderly adults.

## Modeling the relation between motor unit discharge rate and rate of force development

**Figure 1:**
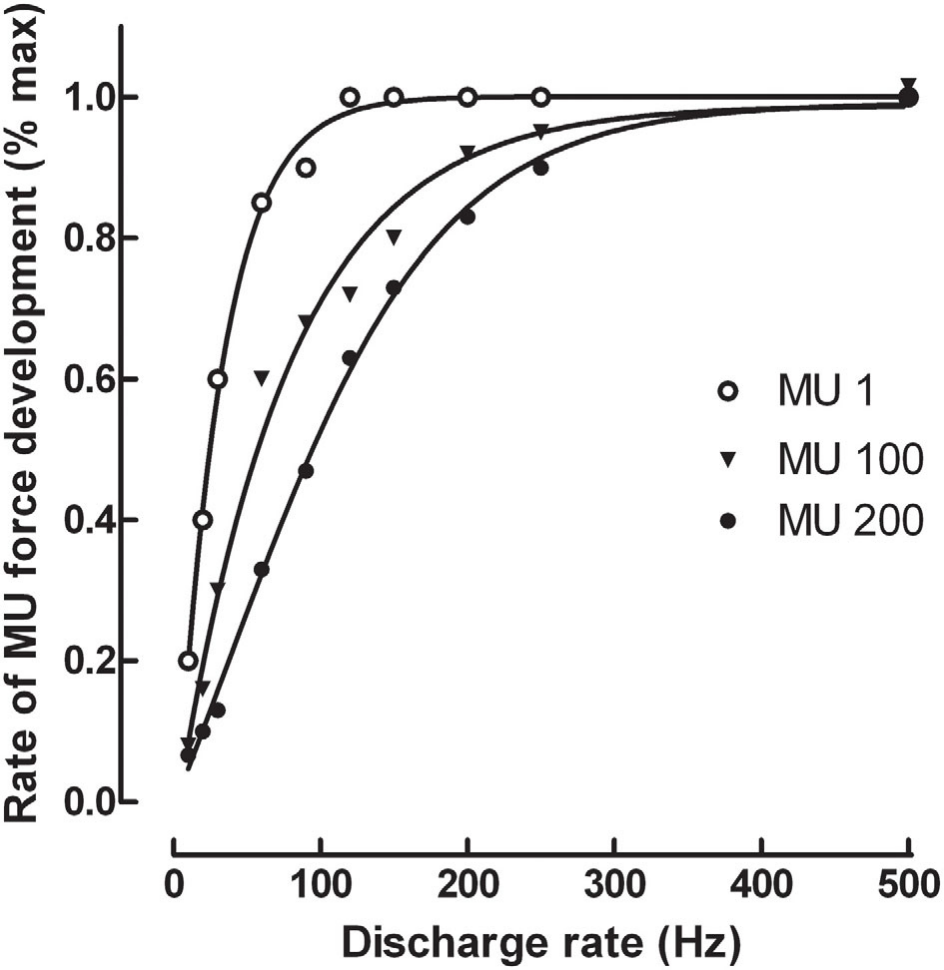
**Simulation of the relation between motor unit discharge rate and maximal rate of force development for the 1st, 100th, and 200th motor unit (MU) of a pool of 200 units in the tibialis anterior muscle**. The simulation was based on a model developed by Fuglevand et al. (1993) with the inclusion of the spike-triggered average forces for motor units published by Van Cutsem et al. (1998). The force generated by each motor unit was simulated for 4 successive discharges generated at constant frequencies ranging from 10 to 500 Hz before the first derivative was computed to obtain the maximal rate of force development.

To further analyse the effect of a change in discharge rate on the maximal rate of force development, isometric force produced by single motor units was simulated from a model that contains a pool of 200 units (Fuglevand et al., 1993; Duchateau and Enoka, 2002). To that purpose, mechanical properties (peak force and time to peak force) of motor units obtained from the spike-triggered averaging method in the tibialis anterior (Van Cutsem et al., 1998) were inserted into the model. Data indicated that an increase in discharge rate up to 100–200 Hz augmented substantially the rate of force development for all units of the pool (Figure 1). Nonetheless, further increase in discharge rate has less influence excepted for the faster units (MU 100 and MU 200) of the pool, reflecting difference in speed-related properties between low- and high threshold motor units. These simulated data underscore the critical role of maximal motor unit discharge rate on the ability to rapidly develop force.

## Concluding remarks

Together, experimental and simulated data indicate that a high initial motor unit discharge rate at the onset of a fast contraction plays a critical role to reach a high rate of force development. Furthermore, and because the instantaneous discharge rates of motor units at the onset of ballistic contractions are much greater than those recorded during slow contractions and not yet influenced by history-dependent effects, ballistic contractions from a resting state can be used to assess the maximal motor neuron discharge rate in human. Nonetheless, as the acquisition of a simple motor task such as index finger abduction requires up to ~300 repetitions to reach maximal acceleration capability (Lee et al., 2010), subjects must be familiarized beforehand with ballistic contractions of the muscle under study.

### Conflict of interest statement

The authors declare that the research was conducted in the absence of any commercial or financial relationships that could be construed as a potential conflict of interest.
